# Vascular Notch-Related Protein Expression in a Rat Model of Central Venous Catheter-Associated *Candida albicans* Infection Under Antifungal and Prostaglandin-Pathway Interventions

**DOI:** 10.3390/pathogens15070748

**Published:** 2026-07-17

**Authors:** Hande Berk Cam, Leyla Kilinc, Hasan Huseyin Avci, Hakan Soylu, Tugrul Cakir, Derya Seyman, Filiz Kizilates, Nefise Oztoprak, Ismail Ustunel

**Affiliations:** 1 Infectious Diseases and Clinical Microbiology Clinic, Antalya Training and Research Hospital, Antalya 07100, Turkey; filizkizilates@gmail.com; 2 Department of Histology and Embryology, Akdeniz University Medical School, Antalya 07070, Turkey; leylakilinc5561@gmail.com (L.K.); iustunel@akdeniz.edu.tr (I.U.); 3 Department of Family Medicine, Akdeniz University Medical School, Antalya 07070, Turkey; hasanavci@akdeniz.edu.tr; 4 Department of Histology and Embryology, Düzce University Medical School, Düzce 81620, Turkey; hakansoylu@duzce.edu.tr; 5 General Surgery Clinic, Antalya Training and Research Hospital, Antalya 07100, Turkey; tugrul.cakir@alanyauniversity.edu.tr; 6 Department of Infectious Diseases and Clinical Microbiology, University of Health Sciences, Antalya Training and Research Hospital, Antalya 07100, Turkey; derya.seyman@sbu.edu.tr (D.S.); nefiseoztoprak.cuvalci@sbu.edu.tr (N.O.)

**Keywords:** *Candida albicans*, Notch signaling, central venous catheter infection, antifungal therapy, prostaglandin pathway, immunohistochemistry

## Abstract

Central venous catheters are a major risk factor for *Candida albicans* vascular infections, which remain challenging to manage. Although antifungal therapy is standard, the host pathways shaping vascular responses—particularly the Notch signaling pathway (NSP)—are not well characterized in this context. In addition, the potential influence of the prostaglandin pathway on vascular NSP-related responses during infection remains unclear. In this study, a rat model of central venous catheter-associated *C. albicans* infection was used to evaluate microbiological outcomes and vascular NSP-related protein expression. Immunohistochemical analyses were performed to assess *Candida* immunostaining alongside the expression of Notch receptors (Notch1–3) and ligands (DLL1/4, Jagged1/2) in vascular tissues. Experimental groups included sham, infected control, antifungal-treated (fluconazole, caspofungin, liposomal amphotericin B), and prostaglandin pathway-intervention groups (sulprostone and sulprostone followed by indomethacin). *C. albicans* infection was associated with higher vascular NSP-related protein expression compared with sham animals. Antifungal-treated groups showed lower NSP-related protein expression, while fungicidal agents were associated with absence of fungal growth in catheter and kidney cultures. In the sulprostone–indomethacin-treated group, NSP-related protein expression levels were lower than those in the sulprostone-treated group despite persistent fungal burden. In conclusion, central venous catheter-associated *C. albicans* infection was associated with altered vascular NSP-related protein expression. Differences in NSP-related protein expression patterns were observed across antifungal- and prostaglandin pathway-intervention groups. These findings are descriptive and do not allow causal inference but may provide a basis for future studies exploring the role of NSP in vascular responses to *C. albicans* infection.

## 1. Introduction

Healthcare-associated *Candida* infections remain a major cause of morbidity and mortality in hospitalized patients, with reported case fatality rates approaching 40% despite advances in antifungal therapy [1,2,3,4]. *Candida albicans* is among the most common causes of invasive fungal disease in humans [1,2,3,4]. *C. albicans* possesses several virulence attributes that contribute to catheter-associated infection, including adhesion to biomaterial surfaces, biofilm formation, hyphal morphogenesis, secretion of hydrolytic enzymes, tissue invasion, and host cell damage mediated by candidalysin [5,6,7]. These virulence determinants facilitate colonization of central venous catheters, host tissue damage, persistence within biofilms, and bloodstream infection, making *C. albicans* a clinically important pathogen in catheter-related candidiasis [5,6,7]. Central venous catheters (CVCs) are the predominant source of healthcare-associated candidemia, largely due to biofilm formation on intravascular surfaces, which enables persistent colonization and contributes to therapeutic failure [2,8].

Fluconazole, caspofungin, and liposomal amphotericin B are widely used in *Candida* infections. While fluconazole acts as a fungistatic agent, echinocandins and amphotericin B formulations exert fungicidal activity [2,8]. However, recurrent infection and the limitations of pathogen-directed treatment highlight the need for complementary strategies that also target host-driven components of disease.

Among such host factors, prostaglandin E_2_ (PGE_2_) has emerged as a relevant mediator of *Candida* morphogenesis and pathogenicity [9,10,11]. Experimental studies show that nonsteroidal anti-inflammatory drugs (NSAIDs), which inhibit PGE_2_ synthesis, can influence fungal behavior and host responses, suggesting a potential role for host-directed adjunctive therapies [12,13,14]. However, the relationship between prostaglandin pathway interventions and vascular host signaling responses during *Candida* infection remains incompletely characterized in vivo.

The Notch signaling pathway (NSP) is a conserved cell–cell communication system that regulates immune function, endothelial biology, and vascular homeostasis [15,16,17]. Notch receptors (Notch1–4) and their ligands (DLL1, DLL4, Jagged1, Jagged2) participate in host–pathogen interactions across bacterial, viral, and parasitic infections, including tuberculosis, sepsis, influenza, and malaria [17,18,19,20,21]. In contrast, the role of NSP in fungal disease remains poorly defined. Prior evidence is limited to models involving *Histoplasma capsulatum*, *Cryptococcus neoformans*, *Paracoccidioides brasiliensis*, and *Pneumocystis* spp. [22,23,24,25,26,27], and to the best of our current knowledge, studies examining Notch pathway expression in *C. albicans* infection remain very limited. In addition, extensive evidence supports a central role for Notch signaling in vascular and endothelial cell biology, underscoring its potential relevance to host vascular responses during infection [28,29].

To address this gap, we investigated vascular Notch-related protein expression in a rat model of CVC-associated *C. albicans* infection. We evaluated Notch-related protein expression patterns in animals receiving standard antifungal therapy and assessed, in an exploratory manner, whether pharmacologic interventions targeting the PGE_2_/COX axis were associated with changes in vascular Notch-related protein expression in vivo. Overall, this work is descriptive and hypothesis-generating, aiming to characterize vascular Notch expression patterns across experimental conditions rather than to establish mechanistic or causal relationships.

Although four Notch receptors have been described in mammals, the present study focused on Notch1–3 and their canonical ligands, as these components have been most consistently implicated in infection-associated inflammatory and endothelial activation. Notch4 was not included in the biomarker panel because emerging evidence suggests that it may exert context-dependent regulatory or anti-inflammatory functions, rather than promoting inflammatory activation in infection-related settings [30].

## 2. Materials and Methods

A controlled, randomized in vivo model of CVC-associated *C. albicans* infection was used as previously described [31]. Forty-two male Wistar rats (350–400 g) were housed under standard laboratory conditions with free access to food and water. The predefined primary endpoint was microbiological confirmation of infection for model validation, whereas the main analytical focus was on vascular Notch-related protein expression. *C. albicans* ATCC 10231 strain was selected as a well-characterized reference strain that has been widely used in studies investigating *Candida* virulence, biofilm formation, antifungal susceptibility, and experimental infection models [32,33,34]. Detailed procedural descriptions, including catheter preparation, inoculum generation, immunohistochemistry protocols, microbiological workflow, and image analysis, are provided in the Supplementary Material.

Animals were randomly assigned to seven groups (*n* = 6 per group):

Sham (surgical control): Jugular catheter insertion without *C. albicans* (American Type Culture Collection, Manassas, VA, USA) inoculation.

*Candida* control (infected control): Catheter insertion with *C. albicans* inoculation; no treatment.

Fluconazole (FCZ) (Triflucan; Pfizer, Istanbul, Turkey): 10 mg/kg/day IV, administered from 48 h to 120 h post-inoculation (Triflucan, Pfizer).

Caspofungin (CasF) (Merck Sharp & Dohme, Istanbul, Turkey): 5 mg/kg/day IV, administered from 48 h to 120 h post-inoculation (Cancidas, Merck Sharp & Dohme).

Liposomal amphotericin B (LAmB) (AmBisome; Gilead Sciences, Istanbul, Turkey): 4 mg/kg/day IV, administered from 48 h to 120 h post-inoculation (AmBisome, Gilead Sciences).

Sulprostone (SP) (Cayman Chemical, Ann Arbor, MI, USA): 100 µg/day IV, administered from 4 h to 48 h post-inoculation.

Sulprostone + Indomethacin (SP-IND) (Endol; DEVA Holding A.S., Istanbul, Turkey): SP from 4 h to 48 h post-inoculation, followed by IND 3 mg/kg/day orally (PO) from 48 h to 72 h post-inoculation.

Twenty-four hours after catheterization, CVCs were filled with 650 µL of *C. albicans* ATCC 10231 suspension (0.5 McFarland, 1–5 × 10^6^ CFU/mL) and incubated intraluminally for 4 h, then flushed into circulation. Catheters were subsequently locked with heparinized saline (100 U/mL). Infection was allowed to progress for 48 h prior to therapy initiation.

Because the study addressed two distinct biological questions, treatment schedules differed across groups. In the SP and SP-IND arms, pharmacologic interventions were initiated early—4 h after inoculation—to explore associations between prostaglandin pathway interventions and Notch-related protein expression. SP was administered from 4 h to 48 h post-inoculation, and in the SP-IND arm, indomethacin was introduced for 24 h, beginning 24 h after the final SP dose. Neither group received antifungal therapy.

In contrast, antifungal treatment (FCZ, CasF, LAmB) was intentionally delayed until 48 h post-inoculation to allow the infection to become established [31]. Antifungal therapy was then continued for 72 h.

**Figure 1:**
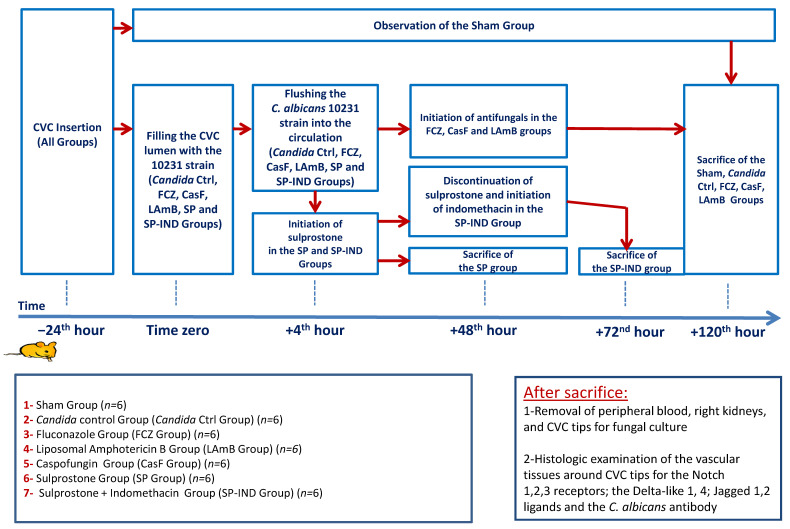
Study timeline and experimental design. Schematic overview of the rat central venous catheter (CVC) model of *Candida albicans* infection. All animals underwent right internal jugular vein catheterization. *C. albicans* inoculation was performed 24 h after catheter insertion, followed by intraluminal incubation for 4 h and flushing into the circulation. Antifungal therapy (fluconazole, caspofungin, liposomal amphotericin B) was initiated 48 h after inoculation and administered for 72 h. Host-directed modulation groups received sulprostone (SP) initiated 4 h after inoculation for 48 h, or SP for 48 h followed by indomethacin (SP-IND) for an additional 24 h. Animals were sacrificed at 48 h (SP), 72 h (SP-IND), or 120 h (Sham, *Candida* control, antifungal-treated groups).

As a result of these design differences, animals were sacrificed at different post-inoculation time points: 48 h for the SP group, 72 h for the SP-IND group, and 120 h for the Sham, Candida control, FCZ, CasF, and LAmB groups (Figure 1).

At sacrifice, catheter tips, peripheral blood, and right kidneys were aseptically collected. Samples were cultured on Sabouraud dextrose agar, and blood cultures were incubated for up to 10 days. All microbiological analyses were performed blinded to group allocation.

Antifungal susceptibility testing was performed using the Sensititre YeastOne YO10 system according to the manufacturer’s instructions as part of routine laboratory workflow. Minimal inhibitory concentrations (MICs) obtained with this system were subsequently interpreted using European Committee on Antimicrobial Susceptibility Testing (EUCAST) clinical breakpoints available at the time of analysis [35]. Susceptibility testing was included as part of the microbiological characterization and validation of the experimental model, rather than as a primary antifungal susceptibility endpoint. Detailed baseline MIC values of the inoculated *Candida albicans* ATCC 10231 strain and MIC values of isolates recovered from culture-positive catheter (CVC) and kidney tissues are provided in Supplementary Table S1.

Formalin-fixed, paraffin-embedded vascular tissues were stained using anti-*Candida* antibodies, as well as antibodies against DLL1/4, Jagged1/2, and Notch1–3. Immunohistochemical staining intensity was assessed by analyzing three distinct images from three randomly selected animals per group using ImageJ/Fiji software version 1.52 (National Institutes of Health, Bethesda, MD, USA). For each selected animal, three high-magnification field images captured at 40× magnification were analyzed. After subtracting background staining, the amount of DAB-positive staining was calculated, and the mean value derived from the three images was used as the percentage value representing that animal. To ensure consistency across samples, all evaluations were performed by a single experienced researcher; therefore, inter-rater variability analysis was not conducted. Full staining protocols and antibody specifications are provided in Supplementary File S1 (Supplementary Methods).

The experimental design and sacrifice schedule are shown in Figure 1.

Group comparisons were performed using one-way ANOVA with Sidak’s post hoc test. However, given differences in sacrifice time points across groups, these analyses were considered exploratory and should be interpreted with caution. *p* < 0.05 was considered statistically significant. Statistical analyses were performed using GraphPad Prism 8 (GraphPad Software, San Diego, CA, USA).

## 3. Results

All predefined endpoints were successfully assessed in all animals. The following subsections describe (i) microbiological outcomes from CVC, blood, and kidney cultures and (ii) vascular *Candida* immunostaining and Notch-related protein expression. As outlined in the Materials and Methods, statistical comparisons should be interpreted as exploratory due to differences in sacrifice time points across groups.

### 3.1. Microbiological Findings

The blood cultures were negative across all groups. In contrast, *C. albicans* growth was consistently detected in CVC and kidney cultures from the *Candida* control, FCZ, SP, and SP-IND groups, indicating the presence of infection at catheter and renal tissue sites despite negative blood cultures. No fungal growth was observed in either CVC or kidney specimens from the CasF and LAmB groups, revealing microbiological sterilization at these sites. The Sham group showed no fungal growth in any specimen.

### 3.2. Candida Immunostaining

*Candida* immunostaining was highest in the *Candida* control group, with prominent staining in vascular and muscle tissues, and significantly higher than in the Sham group and all antifungal-treated groups (adjusted *p* < 0.0001). The Sham group showed minimal staining. All antifungal groups (FCZ, CasF, LAmB) showed significantly lower expression levels compared with the *Candida* control group (adjusted *p* < 0.0001).

**Figure 2:**
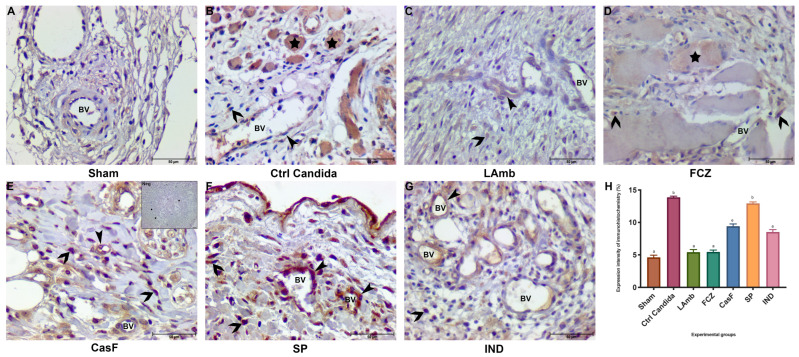
*Candida* immunostaining in internal jugular vein tissues. Representative *Candida* immunohistochemistry (IHC) images and ImageJ-based quantitative analysis of *Candida* immunostaining intensity across all groups. B.V.: blood vessel; black stars: muscle; wide arrowhead: stromal cell; narrow arrowhead: endothelium; LAmB: liposomal amphotericin-B; FCZ: fluconazole; CasF: caspofungin; SP: sulprostone; IND: sulprostone + indomethacin. Groups: (**A**) Sham, (**B**) *Candida* control, (**C**) LAmB, (**D**) FCZ, (**E**) CasF, (**F**) SP, (**G**) IND, (**H**) Quantitative analysis of immunohistochemical staining intensity (%) across the experimental groups. Different superscript letters denote statistically significant differences (adjusted *p* < 0.05).

*Candida* immunostaining in the sulprostone (SP) group was localized to blood vessels and endothelium. Expression levels showed no statistically significant difference compared with the *Candida* control group (adjusted *p* > 0.05) and were significantly higher than in the Sham and antifungal-treated groups (adjusted *p* < 0.0001). In contrast, the sulprostone–indomethacin (SP-IND) group showed significantly lower *Candida* immunostaining compared with both the *Candida* control and SP groups (adjusted *p* < 0.0001) (Figure 2).

### 3.3. Notch Ligand Expression (DLL1, DLL4, Jagged1, Jagged2)

All ligands (DLL1, DLL4, Jagged1, Jagged2) showed higher expression levels in the *Candida* control group, with significantly higher expression compared with the Sham and all antifungal-treated groups (adjusted *p* < 0.0001). Staining was primarily localized to vascular and muscle tissues in the *Candida* control group. Lower ligand expression levels were observed in antifungal-treated groups compared with the *Candida* control group (adjusted *p* < 0.0001), with the lowest values in the CasF and LAmB groups. The LAmB group showed the lowest DLL1 and Jagged1 levels, whereas slightly higher values were observed for some ligands in the CasF group.

Ligand expression levels (DLL1, DLL4, Jagged1, Jagged2) in the sulprostone (SP) group showed no statistically significant difference compared with the *Candida* control group (adjusted *p* > 0.05). Expression levels in the SP group were higher than in the Sham and antifungal-treated groups (adjusted *p* < 0.0001). In contrast, lower expression levels across all ligands were observed in the sulprostone–indomethacin (SP-IND) group compared with both the *Candida* control and SP groups; however, these comparisons are limited by differences in sacrifice time points (adjusted *p* < 0.0001) (Supplementary Figures S1–S4).

### 3.4. Notch Receptor Expression (Notch1, Notch2, Notch3)

Notch receptor (Notch1, Notch2, Notch3) expression patterns were similar across groups, with the highest levels observed in the *Candida* control group and the lowest levels in Sham animals. Lower expression levels were observed in all antifungal-treated groups compared with the *Candida* control group (adjusted *p* < 0.0001), with the lowest Notch1 and Notch3 values observed in the liposomal amphotericin B group. In the *Candida* control group, Notch1 receptor expression was prominent in muscle tissues, whereas stromal tissue involvement was notable for Notch2 receptors.

In the SP group, Notch1 and Notch2 receptor expression was mainly detected in muscle tissues, whereas blood vessel involvement was more prominent for the Notch3 receptor. Receptor expression levels in the SP group showed no statistically significant difference compared with the *Candida* control group (adjusted *p* > 0.05). Expression levels in the SP group were higher than in the Sham and antifungal-treated groups (all adjusted *p* < 0.01), with the exception of Notch2 expression in the caspofungin-treated group (adjusted *p* > 0.05). In contrast, lower expression levels for all Notch receptors were observed in the SP-IND group compared with both the *Candida* control and SP groups; however, these comparisons are limited by differences in sacrifice time points (adjusted *p* < 0.0001) (Supplementary Figures S5–S7).

## 4. Discussion

The Notch signaling pathway (NSP) is involved in immune regulation and vascular homeostasis and has been extensively studied in bacterial and viral sepsis models. However, its role in fungal disease—particularly *C. albicans* infection—remains incompletely characterized, with only limited evidence derived from models involving non-*Candida* fungal infections [22,23,24,25,26]. The present study contributes to this area by providing in vivo data describing vascular Notch-related protein expression patterns in a rat model of CVC-associated *C. albicans* infection.

In this model, *C. albicans* infection was associated with higher expression of Notch receptors (Notch1–3) and ligands (DLL1, DLL4, Jagged1, Jagged2) within vascular tissues, accompanied by prominent vascular and perivascular *Candida* immunostaining. In contrast, Sham animals exhibited minimal expression. These expression patterns resemble observations reported in bacterial sepsis models, where endothelial and immune cell Notch components have been described alongside inflammatory responses [18,36,37], suggesting that vascular Notch signaling may be involved in host responses to invasive infection.

A central observation of this study is that NSP-related protein expression patterns varied across experimental conditions and were associated with different treatment groups. Lower expression levels of Notch receptors and ligands were observed in antifungal-treated animals compared with *Candida* control animals. Because the reference *C. albicans* strain used in this study was fully susceptible to all antifungal agents, the absence of fungal growth in fungicidal treatment groups was consistent with the known susceptibility profile of the strain and primarily served to validate the experimental model rather than to evaluate antifungal efficacy.

Notably, fluconazole treatment was associated with lower vascular Notch-related protein expression despite persistent fungal burden. The absence of microbiological sterilization in tissues in the fluconazole-treated group in this model may reflect its fungistatic activity and/or the limited duration of treatment [38,39]. This dissociation between lower NSP-related protein expression and persistent fungal burden may reflect the complexity of host signaling responses during antifungal treatment.

PGE_2_ has been linked to fungal virulence in earlier studies, particularly by enhancing morphologic transitions and pathogenicity [10,40]. In addition, several in vitro studies have suggested that nonsteroidal anti-inflammatory drugs (NSAIDs) can influence host–pathogen interactions and have been associated with changes in endothelial signaling [12,13,14]. This background provided the biological rationale for including prostaglandin pathway-targeting interventions in the present study, which focused on characterizing vascular Notch-related protein expression patterns during fungal infection rather than assessing antifungal efficacy or virulence per se. In our model, animals in the sulprostone (SP) group showed no statistically significant difference in Notch-related protein expression and *Candida* immunostaining compared with the *Candida* control group. However, because these animals were sacrificed at an earlier time point (48 h) than the *Candida* control group (120 h), and no infected control was included at the corresponding early time point, the relative contribution of prostaglandin-related influences versus early infection dynamics cannot be clearly distinguished.

In the sulprostone–indomethacin (SP-IND) group, Notch-related protein expression levels were significantly lower than those observed in both the SP and *Candida* control groups. Nevertheless, given the absence of time-matched infected controls and the known pleiotropic effects of NSAIDs, these findings are interpreted as descriptive and hypothesis-generating. Future studies incorporating time-matched controls and mechanistic approaches will be required to further explore the relationship between prostaglandin pathway-targeting interventions and vascular host signaling during *Candida* infection.

NSP has been implicated in immune-cell maturation, cytokine responses, and endothelial activation [41,42]. The predominantly vascular localization observed in our study raises the possibility that Notch signaling may be involved in endothelial responses during invasive *Candida* infection. Whether these expression changes contribute to vascular dysfunction or reflect downstream inflammatory signaling cannot be determined from the current data and warrants further mechanistic investigation.

Several limitations should be acknowledged. Differences in sacrifice time points may have introduced temporal bias, and the model rarely produces candidemia. Accordingly, the findings should be interpreted in the context of localized catheter-associated infection rather than disseminated or systemic immune responses. In addition, interspecies differences in endothelial signaling and immune regulation may affect the direct translational relevance of these findings to human vascular biology. Furthermore, the present study employed a single reference strain (*C. albicans* ATCC 10231); therefore, the findings should be interpreted within the context of this strain, as substantial genetic and phenotypic diversity among *C. albicans* isolates may influence virulence-associated characteristics. Indomethacin may exert biological effects beyond cyclooxygenase inhibition, which were not specifically examined in this study; therefore, the mechanisms underlying the observed Notch-related protein expression patterns cannot be attributed to COX inhibition alone. Measurement of downstream inflammatory markers such as IL-6 or TNF-α in vascular tissues was not performed and could provide additional insight in future studies to better link Notch-related protein expression patterns with inflammatory and endothelial responses. Additionally, the use of one-way ANOVA across groups evaluated at different time points should be interpreted with caution, as temporal changes in Notch-related protein expression may confound group comparisons. Functional experiments addressing the biological consequences of changes in Notch-related protein expression, such as effects on host survival, cytokine responses, or vascular integrity, were beyond the scope of this study. Future studies incorporating analysis of canonical Notch target genes (e.g., Hes1, Hey1) and/or endothelial functional assays would be important to further define the biological significance of the observed Notch-related protein expression patterns. Despite these limitations, the findings may provide new descriptive insight into vascular Notch signaling during *Candida* infection and offer a framework for future mechanistic and translational studies.

## 5. Conclusions

In conclusion, CVC-associated *C. albicans* infection was associated with higher Notch-related protein expression in vascular tissues in this rat model. Differences in vascular Notch-related protein expression patterns were observed across antifungal-treated, sulprostone-treated, and sulprostone–indomethacin-treated groups; however, these findings are observational and should be interpreted with caution in light of differences in sacrifice timing and the absence of time-matched infected controls. Overall, the results suggest that vascular Notch signaling may be involved in host responses during invasive *Candida* infection and that its expression patterns can vary independently of fungal burden. Further studies incorporating time-matched controls and functional assays are required to further define the biological significance of these observations and to explore the relationship between this pathway and host signaling responses during fungal infection.

## Data Availability

The original contributions presented in this study are included in the article and Supplementary Material. Further inquiries can be directed to the corresponding author.

## Supplementary Materials

The following supporting information can be downloaded at: https://www.mdpi.com/article/10.3390/pathogens15070748/s1.

## Abbreviations

The following abbreviations are used in this manuscript:

ATCC	American Type Culture Collection
CasF	Caspofungin
CFU	Colony-forming unit
COX	Cyclooxygenase
CVC	Central venous catheter
DLL1	Delta-like ligand 1
DLL4	Delta-like ligand 4
EUCAST	European Committee on Antimicrobial Susceptibility Testing
FCZ	Fluconazole
h	Hour
IHC	Immunohistochemistry
IND	Indomethacin
IV	Intravenous
Jag1	Jagged1
Jag2	Jagged2
LAmB	Liposomal amphotericin B
mg	Milligram
mL	Milliliter
MIC	Minimum inhibitory concentration
µL	Microliter
NSAIDs	Nonsteroidal anti-inflammatory drugs
NSP	Notch signaling pathway
Notch1	Notch receptor 1
Notch2	Notch receptor 2
Notch3	Notch receptor 3
*p*	Probability value (*p*-value)
PG	Prostaglandin
PGE_2_	Prostaglandin E_2_
PO	Per os (oral administration)
SP	Sulprostone
U	Unit
YO10	Sensititre YeastOne YO10 microdilution system
