# Supplementary material for: Vascular Notch-Related Protein Expression in a Rat Model of Central Venous Catheter-Associated Candida albicans Infection Under Antifungal and Prostaglandin-Pathway Interventions
Source: Pathogens. 2026 Jul 17;15(7):748. doi: 10.3390/pathogens15070748 (PMC13415132; doi:10.3390/pathogens15070748)
Supplement: Supplementary file 1 [file pathogens-15-00748-s001.zip › Supplementary Figures (S1–S7).pdf]

Supplementary Figures (S1–S7)

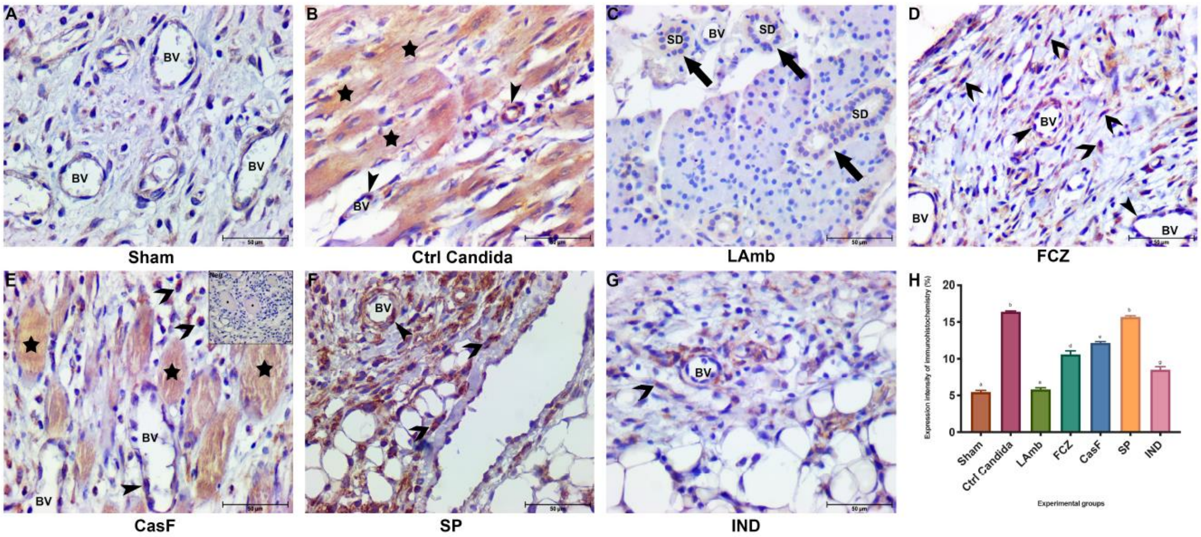

Figure S1. Vascular expression of DLL1 in internal jugular vein tissues. Representative immunohistochemistry (IHC) images and ImageJ-based quantitative analysis of staining intensity across all groups. B.V.: blood vessel; black stars: muscle; wide arrowhead: stromal cell; narrow arrowhead: endothelium; long arrow: secretory duct cell; SD: secretory duct; LAmB: liposomal amphotericin B; FCZ: fluconazole; CasF: caspofungin; SP: sulprostone; IND: sulprostone + indomethacin. (A) Sham; (B) Candida control; (C) LAmB; (D) FCZ; (E) CasF; (F) SP; (G) IND. Different superscript letters indicate statistically significant differences (adjusted P < 0.05).

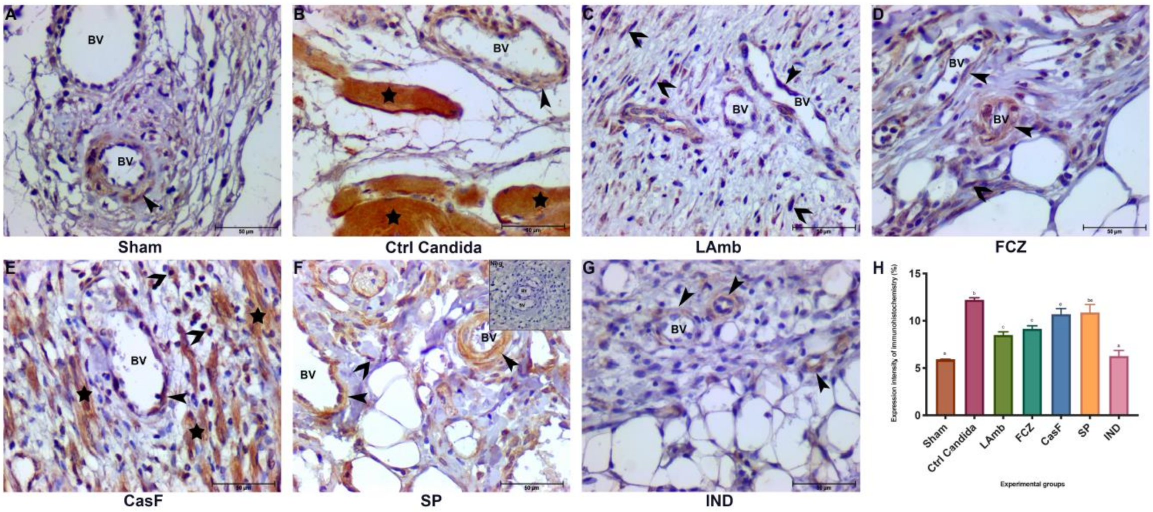

Figure S2. Vascular expression of DLL4 in internal jugular vein tissues. Representative immunohistochemistry (IHC) images and ImageJ-based quantitative analysis of staining intensity across all groups. B.V.: blood vessel; black stars: muscle; wide arrowhead: stromal cell; narrow arrowhead: endothelium; LAmB: liposomal amphotericin B; FCZ: fluconazole; CasF: caspofungin; SP: sulprostone; IND: sulprostone + indomethacin. (A) Sham; (B) Candida control; (C) LAmB; (D) FCZ; (E) CasF; (F) SP; (G) IND. Different superscript letters indicate statistically significant differences (adjusted P < 0.05).

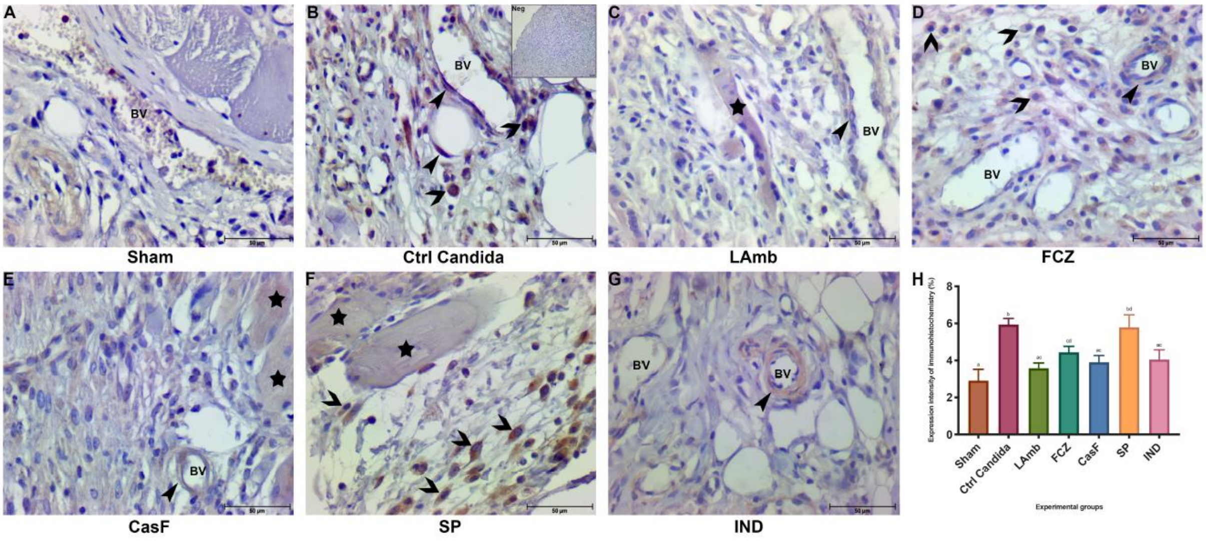

Figure S3. Vascular expression of Jagged1 in internal jugular vein tissues. Representative immunohistochemistry (IHC) images and ImageJ-based quantitative analysis of staining intensity across all groups. B.V.: blood vessel; black stars: muscle; wide arrowhead: stromal cell; narrow arrowhead: endothelium; LAmB: liposomal amphotericin B; FCZ: fluconazole; CasF: caspofungin; SP: sulprostone; IND: sulprostone + indomethacin. (A) Sham; (B) Candida control; (C) LAmB; (D) FCZ; (E) CasF; (F) SP; (G) IND. Different superscript letters indicate statistically significant differences (adjusted P < 0.05).

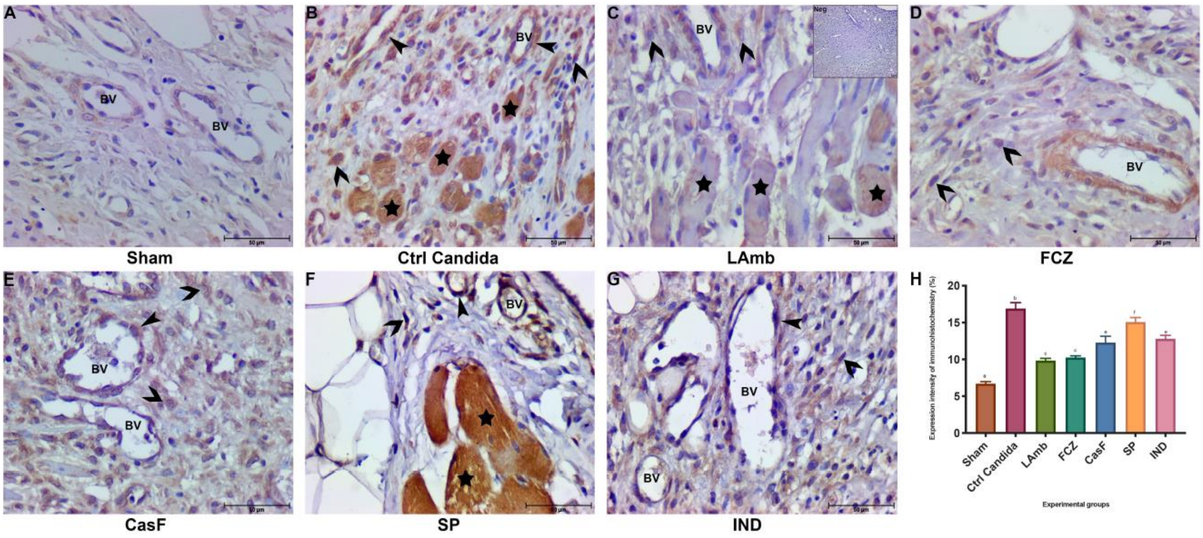

Figure S4. Vascular expression of Jagged2 in internal jugular vein tissues. Representative immunohistochemistry (IHC) images and ImageJ-based quantitative analysis of staining intensity across all groups. B.V.: blood vessel; black stars: muscle; wide arrowhead: stromal cell; narrow arrowhead: endothelium; LAmB: liposomal amphotericin B; FCZ: fluconazole; CasF: caspofungin; SP: sulprostone; IND: sulprostone + indomethacin. (A) Sham; (B) Candida control; (C) LAmB; (D) FCZ; (E) CasF; (F) SP; (G) IND. Different superscript letters indicate statistically significant differences (adjusted P < 0.05).

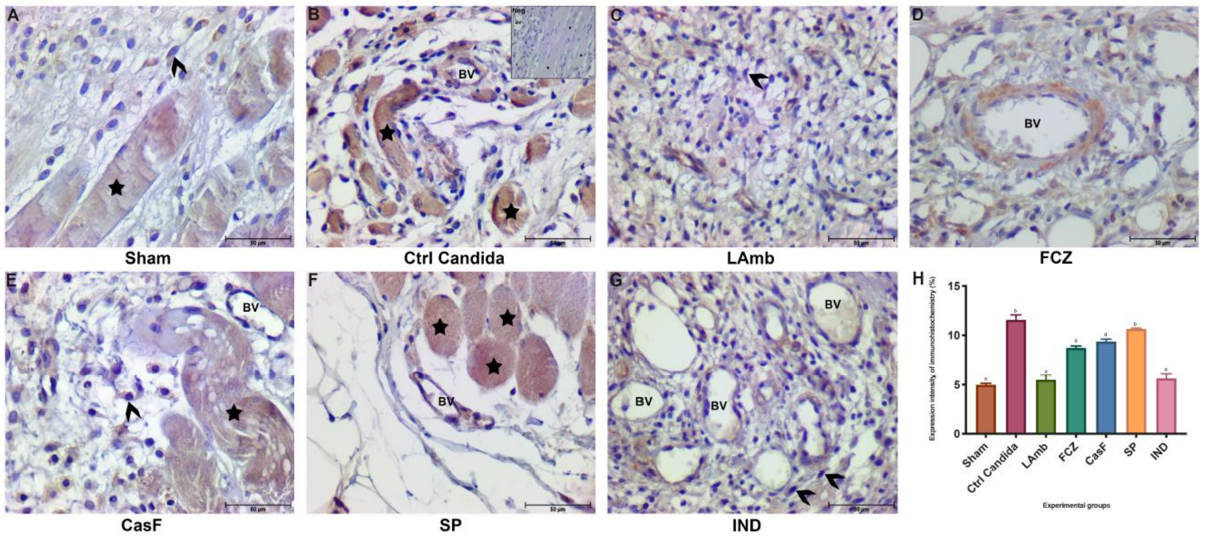

Figure S5. Vascular expression of Notch1 in internal jugular vein tissues. Representative immunohistochemistry (IHC) images and ImageJ-based quantitative analysis of staining intensity across all groups. B.V.: blood vessel; black stars: muscle; wide arrowhead: stromal cell; LAmB: liposomal amphotericin B; FCZ: fluconazole; CasF: caspofungin; SP: sulprostone; IND: sulprostone + indomethacin. (A) Sham; (B) Candida control; (C) LAmB; (D) FCZ; (E) CasF; (F) SP; (G) IND. Different superscript letters indicate statistically significant differences (adjusted P < 0.05).

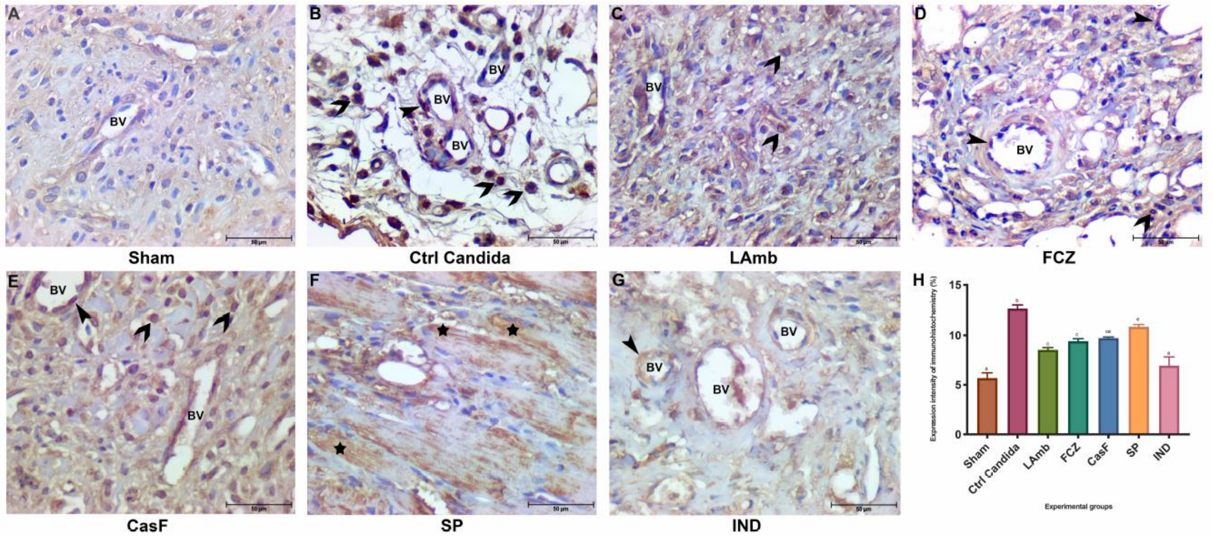

Figure S6. Vascular expression of Notch2 in internal jugular vein tissues. Representative immunohistochemistry (IHC) images and ImageJ-based quantitative analysis of staining intensity across all groups. B.V.: blood vessel; black stars: muscle; wide arrowhead: stromal cell; narrow arrowhead: endothelium; LAmB: liposomal amphotericin B; FCZ: fluconazole; CasF: caspofungin; SP: sulprostone; IND: sulprostone + indomethacin. (A) Sham; (B) Candida control; (C) LAmB; (D) FCZ; (E) CasF; (F) SP; (G) IND. Different superscript letters indicate statistically significant differences (adjusted P < 0.05).

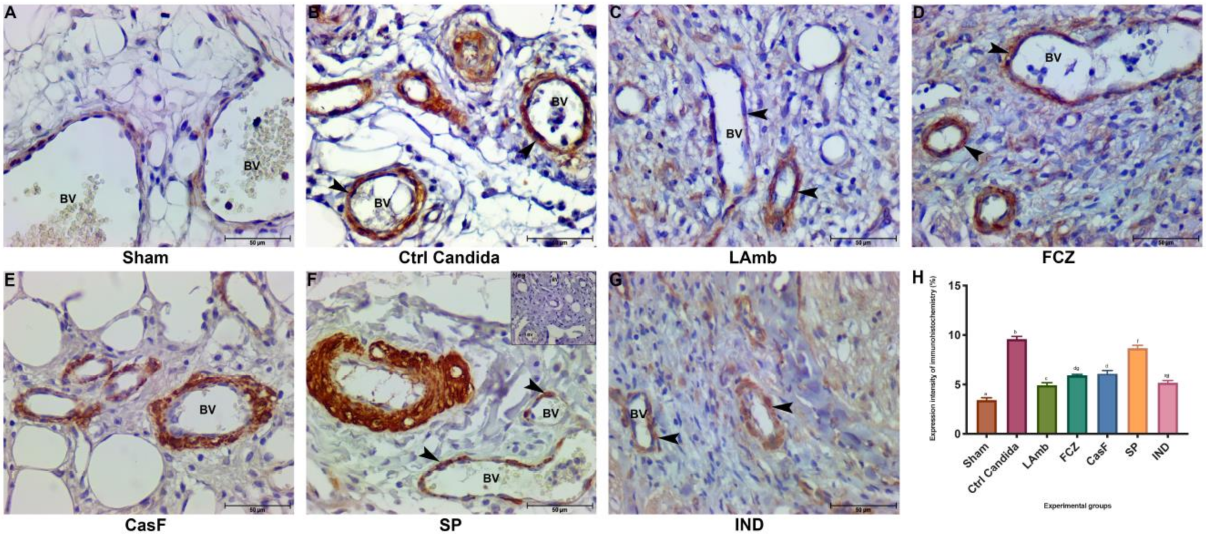

Figure S7. Vascular expression of Notch3 in internal jugular vein tissues. Representative immunohistochemistry (IHC) images and ImageJ-based quantitative analysis of staining intensity across all groups. B.V.: blood vessel; narrow arrowhead: endothelium; LAmB: liposomal amphotericin B; FCZ: fluconazole; CasF: caspofungin; SP: sulprostone; IND: sulprostone + indomethacin. (A) Sham; (B) Candida control; (C) LAmB; (D) FCZ; (E) CasF; (F) SP; (G) IND. Different superscript letters indicate statistically significant differences (adjusted $P < 0.05$).
