# Supplementary material for: Vascular Notch-Related Protein Expression in a Rat Model of Central Venous Catheter-Associated Candida albicans Infection Under Antifungal and Prostaglandin-Pathway Interventions
Source: Pathogens. 2026 Jul 17;15(7):748. doi: 10.3390/pathogens15070748 (PMC13415132; doi:10.3390/pathogens15070748)
Supplement: Supplementary file 1 [file pathogens-15-00748-s001.zip › Supplementary File 1 — Supplementary Methods.pdf]

Supplementary File 1 — Supplementary Methods

Catheter Preparation and Insertion

Polyethylene tubing (inner diameter 0.76 mm, outer diameter 1.52 mm; Intramedic, Becton Dickinson; catalog no. 427430, LOT 5247571) was cut into 50-cm segments, corresponding to an inner volume of approximately 650 μL including the distal stopper. Catheters were sterilized before use.

Under ketamine (80 mg/kg, IP; Lloyd Laboratories) and xylazine (8 mg/kg, IP; AnaSed) anesthesia, a right anterior cervical incision was made and the internal jugular vein was exposed. After a small longitudinal venotomy, the catheter was flushed with heparinized saline (100 U/mL) and advanced 2 cm into the superior vena cava above the right atrium. The catheter was secured with silk sutures, tunneled subcutaneously to the scapular region, and externalized. The distal end was connected to an 18-gauge plastic cannula fixed to the cage wall. Postoperative analgesia was provided with tramadol hydrochloride (12.5 mg/kg, IP). Catheters were flushed daily with 0.9% NaCl containing heparin (100 U/mL).

Candida Inoculum Preparation and Infection Protocol

Candida albicans ATCC 10231 (LOT 162) was cultured on Sabouraud dextrose agar (SDA) at 35 °C for 24 h. Colonies were suspended in sterile pyrogen-free 0.9% NaCl, and turbidity was standardized to 0.5 McFarland (1–5 × 10^6 CFU/mL) using a densitometer (bioSan Den-1B, Latvia). Viability was confirmed by plating on SDA.

Catheterization was performed 24 h before inoculation to allow host protein deposition. Catheters were filled with 650 μL of the inoculum, incubated in situ for 4 h, and then flushed into the circulation to induce infection. Catheters were subsequently locked with heparinized saline (100 U/mL).

Drug Administration

Three antifungal agents were evaluated:
Fluconazole (FCZ) 10 mg/kg/day, IV (Triflucan, Pfizer)
Caspofungin (CasF) 5 mg/kg/day, IV (Cancidas, Merck Sharp & Dohme)
Liposomal amphotericin B (LAmB) 4 mg/kg/day, IV (AmBisome, Gilead Sciences)
Antifungal therapy was initiated 48 h post-inoculation and continued for 72 h.

A prostaglandin E_2 analog group received sulprostone (SP) 100 μg/day IV (Cayman Chemicals, item no. 14765) for 2 days and was sacrificed 48 h after inoculation.

A prostaglandin-suppression group received SP for 2 days, followed by indomethacin (IND) 3 mg/kg/day orally PO (gavage) for 24 h, and was sacrificed at 72 h. IND was administered short-term to minimize nephrotoxicity.

Sham and untreated Candida control groups were sacrificed at 120 h post-inoculation.

Immunohistochemistry

At sacrifice, catheters and perivascular tissues were fixed in neutral-buffered formalin, dehydrated in graded ethanol series, cleared in xylene, and embedded in paraffin. Tissue sections (5 μm) were mounted on glass slides, blocked with normal serum, and incubated with primary antibodies directed against:
Notch1 (Abcam ab52627; 1:300)
Notch2 (Abcam ab8926; 1:100)
Notch3 (Abcam ab23426; 1:100)
DLL1 (Abcam ab84620; 1:50)
DLL4 (Abcam ab7280; 1:100)
Jagged1 (Abcam ab109536; 1:100)
Jagged2 (Abcam ab60041; 1:50)
anti-Candida antibody (Abcam ab53891; 1:600)
Secondary antibodies (Vector BA-1000) were applied at dilutions of 1:250–1:1500. Visualization was performed using DAB chromogen (Sigma D4168), and slides were counterstained with hematoxylin and sealed with Kaiser's glycerol gelatin. Images were acquired using a Zeiss Axioplan microscope (40× magnification).

Microbiological Cultures and Susceptibility Testing

At sacrifice, the intravascular catheter tip (2 cm), peripheral blood, and the right kidney were aseptically collected. Catheter tips were flushed with sterile 0.9% NaCl, vortexed, and plated on SDA (35 °C, 24 h). Blood (100 μL) and kidney homogenates were plated similarly. Cultures negative at 24 h were incubated for up to 10 days.
Antifungal susceptibility testing was performed using Sensititre YeastOne YO10 (TREK Diagnostic Systems; LOTs B7053, B7031, B9203), which includes anidulafungin, amphotericin B, micafungin, caspofungin, flucytosine, posaconazole, voriconazole, itraconazole, and fluconazole. MICs were interpreted using EUCAST clinical breakpoints.
