# Supplementary material for: Vascular Notch-Related Protein Expression in a Rat Model of Central Venous Catheter-Associated Candida albicans Infection Under Antifungal and Prostaglandin-Pathway Interventions
Source: Pathogens. 2026 Jul 17;15(7):748. doi: 10.3390/pathogens15070748 (PMC13415132; doi:10.3390/pathogens15070748)
Supplement: Supplementary file 1 [file pathogens-15-00748-s001.zip › Supplementary Table S1.pdf]

Supplementary Table S1

Supplementary Table S1. Baseline minimal inhibitory concentration (MIC) values of the inoculated *Candida albicans* ATCC 10231 strain and MIC values of isolates recovered from culture-positive catheter (CVC) and kidney tissue cultures.

	MIC values (mg/L)[a]								
	**AND**	**MCF**	**CasF**	**5-FC**	**POS**	**VOR**	**ITRA**	**FCZ**	**AmB**
C. albicans **reference breakpoints**[b]	>0.03	>0.016	-[c]	-[d]	>0.06	>0.25	>0.06	>4	>1
C. albicans **ATCC 10231 strain in vitro MIC Value**	0.015	≤0.008	0.06	≤0.06	0.03	0.015	0.03	0.5	0.25
Candida **control group Mean MIC values (CVC)**	0.038	0.017	0.08	0.08	0.035	0.018	0.06	0.75	0.417
Candida **control group Mean MIC values (Kidney)**	0.035	0.013	0.065	0.07	0.033	0.016	0.055	0.667	0.375
**Fluconazole group Mean MIC values (CVC)**	0.038	0.014	0.07	0.06	0.03	0.018	0.045	0.833	0.292
**Fluconazole group Mean MIC values (Kidney)**	0.038	0.012	0.07	0.06	0.03	0.018	0.04	0.667	0.292
**Sulprostone group Mean MIC values (CVC)**	0.035	0.008	0.06	0.143	0.038	0.015	0.08	0.5	0.292
**Sulprostone group Mean MIC values (Kidney)**	0.04	0.008	0.06	0.08	0.043	0.015	0.09	0.5	0.292
**Indomethacin group Mean MIC values (CVC)**	0.02	0.008	0.03	0.06	0.015	0.012	0.025	0.375	0.25
**Indomethacin group Mean MIC values (Kidney)**	0.03	0.008	0.035	0.06	0.018	0.012	0.038	0.375	0.185

AND: Anidulafungin**, MCF:** Micafungin**, CasF** : Caspofungin**, 5-FC:** 5-Flucytosine**, POS:** Posaconazole**, VOR:** Voriconazole**, ITRA:** Itraconazole**, FCZ:** Fluconazole**, AmB:** Amphotericin-B

[a] MICs were determined using the Sensititre YeastOne YO10 system according to the manufacturer's instructions and interpreted using EUCAST clinical breakpoints (version 10.0, 2020).
[b] EUCAST clinical breakpoints defining the resistant category for *C. albicans* are provided for reference.
[c] Isolates that are susceptible to anidulafungin and micafungin are considered susceptible to caspofungin according to EUCAST antifungal clinical breakpoints 2020 guideline.
[d] EUCAST antifungal clinical breakpoints have not been established for 5-Flucytosine.

Note: No fungal growth was recovered from catheter or kidney tissue cultures in the caspofungin- or liposomal amphotericin B-treated groups; therefore, no recovered isolates from these groups were available for MIC testing.
